# Cr(VI) Sorption/Desorption on Pine Sawdust and Oak Wood Ash

**DOI:** 10.3390/ijerph120808849

**Published:** 2015-07-29

**Authors:** Avelino Núñez-Delgado, María José Fernández-Sanjurjo, Esperanza Álvarez-Rodríguez, Laura Cutillas-Barreiro, JuanCarlos Nóvoa-Muñoz, Manuel Arias-Estévez

**Affiliations:** 1 Department of Soil Science and Agricultural Chemistry, Engineering Polytechnic School, University of Santiago de Compostela, Lugo 27002, Spain; E-Mails: mf.sanjurjo@usc.es (M.J.F.-S.); esperanza.alvarez@usc.es (E.A.-R.); 2 Department of Plant Biology and Soil Science, Faculty of Sciences, Campus Ourense, University of Vigo, Ourense 32004, Spain; E-Mails: lcutillas@uvigo.es (L.C.-B.); edjuanca@uvigo.es (J.C.N.-M.); mastevez@uvigo.es (M.A.-E.)

**Keywords:** chromium, desorption, recycling, sorption, wood by-products

## Abstract

The objective of this work was to study Cr(VI) sorption/desorption on two by-products from the wood industry: pine sawdust and oak wood ash. The retention/release experiments were carried out using standard batch-type trials. In the sorption-phase experiments, pine sawdust showed 23% sorption when a concentration of 100 mg Cr(VI)·L^−1^ was added, whereas sorption on oak wood ash was 17%. In the desorption-phase, chromium release was clearly higher from pine sawdust than from oak wood ash (98% and 66%, respectively). Sorption curves were well fitted to the Freundlich and Lineal models. In view of the results, both materials can be considered of very limited value to remove Cr from polluted soil and water, which can be of relevance regarding its appropriate use as biosorbents and recycled by-products.

## 1. Introduction

Chromium is a heavy metal that can be harmful even at low concentrations [1]. Considering Cr species, Cr(VI) is very toxic and has high solubility, facilitating its mobility in soil and water, whereas Cr(III) is not toxic at low concentrations and has low mobility [2].

Chromium and its compounds originate in the environment mainly from anthropogenic sources such as industry emissions and combustion processes [3]. These authors indicate that the dumping of industrial waste materials significantly increases chromium concentration in soil and is usually accompanied by groundwater contamination. As indicated in Fernández-Calviño *et al.* [4], chromium concentration averages 54 mg · kg^−1^ in natural soils, with higher concentrations in basic and ultrabasic rocks (ranging between 170 and 3400 mg · kg^−1^), giving values up to 200–400 mg · kg^−1^ in soils over serpentinite, as opposed to average Cr concentrations ranging between 12 and 47 mg · kg^−1^ in histosols and sandy soils [5]. Panda and Choudhury [6] note that Cr is a broadline heavy metal, and is phytotoxic either at all concentrations or above certain threshold levels. Fernández-Pazos *et al.* [7] indicate that in the last decades chromium threshold levels for phytotoxicity may have been exceeded in many soils due to anthropogenic sources such as mining and industrial activities, although phosphate fertilizers and organic amendments may be a source of this element. For drinking water, the guideline value is 0.05 mg · L^−1^ for total chromium [8,9].

Different methods can be employed to remove Cr(VI) from polluted waters, such as ionic exchange using polymeric resins [10], or coagulation/flocculation [11]. However, those methods are characterized by high operating costs and generation of sludge rich in heavy metals [12].

Sorption is considered an efficient alternative to remove heavy metals from wastewater [1]. Recently, some researchers have focused on Cr(VI) sorption and bio-sorption from polluted waters [1,13,14,15,16,17,18,19], and Dakiky *et al.* [20] studied low-cost adsorbents to remove Cr(VI) from industrial wastewater. Park *et al.* [21] found that using certain natural biomaterials Cr(VI) was completely removed from the aqueous phase, concluding that the main mechanism affecting Cr(VI) removal was sorption-coupled reduction.

Miretzkya and Fernandez-Cirelli [22] studied the efficacy of lignocellulosic materials (including various types of chemically-modified pine sawdust) to remove chromium from polluted waters. These authors found very different results for the various sawdust they investigated, making evident the convenience of performing research focusing on each individual material to ascertain its efficacy as regards chromium removal.

Fernández-Pazos *et al.* [7] studied Cr(VI) sorption on mussel-shell treated and on unamended forest and vineyard soils, as well as on mussel shell, pyritic material and slate processing fines, finding high Cr(VI) sorption (94%) on the pyritic material, but poor results on all the other substrates (<22%).

Seco-Reigosa *et al.* [23] used oak wood ash to retain arsenic, however the efficacy of this material to remove chromium was not evaluated.

Although many bio-sorbents have been studied previously, to our knowledge no previous work has compared Cr(VI) sorption/desorption on pine sawdust and oak wood ash by means of simultaneous batch-type experiments. In view of that, the main objectives of this research are to determine and compare Cr(VI) sorption/desorption capacities corresponding to pine sawdust and oak wood ash, and to evaluate the fitting to sorption models. These two by-products could be recycled in restoration of degraded environments, such as mine-dumps, thus making especially relevant their pollutant removal potential.

## 2. Material and Methods

### 2.1. Characterization of pine sawdust and oak wood ash

We used commercially available pine sawdust (Vitakraft), and oak wood ash generated in a local bakery oven, in Baleira (Lugo Province, Spain). Both materials were previously studied as regards their arsenic retention capacities [23], and were chemically characterized using standard methods detailed in Seco-Reigosa *et al.* [23]. Table 1 shows the general characteristics of the pine sawdust and oak wood ash used in this work. The pH of the pine sawdust was acidic (4.91), whereas it was alkaline (11.19) for oak wood ash, which showed higher levels for all parameters unless C, exchangeable Al and total Cd.

**Table 1:** General characteristics of the adsorbent materials: oak wood ash and pine sawdust (average values of 3 replicates; the coefficients of variation were <5% in all cases).

Parameter	Oak Ash	Pine Sawdust
pH_water_	11.19	4.91
pH_KCl_	10.57	4.56
C (%)	6.69	46.13
N (%)	0.10	0.03
P_Olsen_ (mg·kg^−1^)	958.9	11.47
Ca_T_ (mg·kg^−1^)	81,031	8087
Mg_T_ (mg·kg^−1^)	24,505	164.4
Na_T_ (mg·kg^−1^)	8095	98.35
K_T_ (mg·kg^−1^)	70,661	540.6
As_T_ (mg·kg^−1^)	4.00	0.39
Cd_T_ (mg·kg^−1^)	0.18	0.39
Cr_T_ (mg·kg^−1^)	66.67	5.19
Cu_T_ (mg·kg^−1^)	590.5	14.87
Ni_T_ (mg·kg^−1^)	51.51	0.00
Zn_T_ (mg·kg^−1^)	728.9	50.82
Al_T_ (mg·kg^−1^)	20.218	260.6
Fe_T_ (mg·kg^−1^)	41.425	234.2
Mn_T_ (mg·kg^−1^)	6778	97.18
Al_ox_ (mg·kg^−1^)	8722	122.5
Fe_ox_ (mg·kg^−1^)	5239	15.62
Ca_e_ (cmol·kg^−1^)	9.81	5.39
Mg_e_ (cmol·kg^−1^)	8.49	1.37
Na_e_ (cmol·kg^−1^)	20.53	0.66
K_e_ (cmol·kg^−1^)	152.44	1.55
Al_e_ (cmol·kg^−1^)	0.00	0.05

X_T_: total concentration of the element; Al_ox_, Fe_ox_: Al and Fe concentrations in ammonium oxalate extract; X_e:_ exchangeable concentration of the element.

### 2.2. Chromium Sorption and Desorption

Chromium sorption trials were performed similarly to that indicated in Fernández-Pazos *et al.* [7]. Firstly, 3 g of solid sample were mixed with 30 mL NaNO_3_ 0.01 M dissolutions containing 0, 0.5, 5, 10, 25, 50 or 100 mg·L^−1^ Cr(VI), prepared from analytical grade K_2_Cr_2_O_7_ (Panreac, Castellar del Vallès, Barcelona, Spain). The suspensions were shaken for 24 h, centrifuged at 4000 rpm for 15 min, and filtered through Whatman acid-washed paper (pore size 2.5 µm). All trials were performed in triplicate. A glass electrode pH-meter (Crison, L’Hospitalet de Llobregat, Barcelona, Spain) was used to measure pH in the equilibrium dissolutions, whereas Cr(total) was determined by Inductively Coupled Plasma Mass Spectrometry (ICP-MS, Varian 820-MS, Palo Alto, CA, USA).

After sorption trials, each sample received 30 mL of a 0.01 M NaNO_3_ solution to facilitate Cr desorption. Each sample was shaken during 24 h, centrifuged and filtered as in the sorption trials [24]. The desorbed Cr and the pH values were determined in all samples, using ICP-MS and pH-meter, respectively.

Cr sorption data were fitted to the Freundlich and Lineal models (Equations (1) and (2), respectively) using the statistical package SPSS 19 (IBM, Armonk, NY, USA).

The Freundlich equation can be formulated as follows:

(1) $$q_{e}=K_{F}\;C_{e}^{n}$$

where *q_e_* (mg·kg^−1^) is the ion sorption per unit of mass of the adsorbent; *C_e_* (mg·L^−1^) is the equilibrium concentration of the dissolved Cr; *K_F_* (L^n^·g^−1^·mg^(1−n)^) is a constant related to the sorption capacity; and *n* (dimensionless) is a constant related to the sorption intensity. The linear equation can be formulated as follows:

(2) $$q_{e}=a\pm K_{d}\;C_{e}$$

where *a* is the ordinate in the origin; and *K_d_* (L·kg^−1^) is the slope of the line, which can be considered equivalent to the coefficient of distribution.

## 3. Results and Discussion

**Figure 1:**
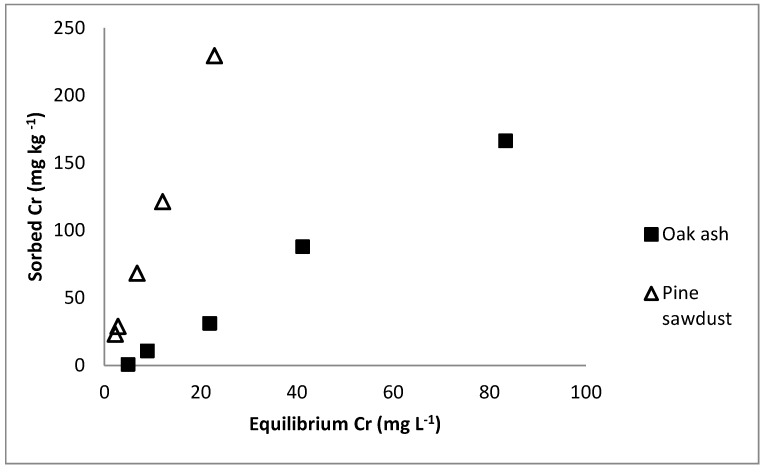
Sorption curves for oak wood ash and pine sawdust.

Figure 1 shows increased Cr sorption as a function of Cr concentration in the equilibrium, with more pronounced slope for pine sawdust, as previously detected for other biosorbents [25,26].

Sorption curves were C-type [27] for both materials, with rather constant slope when increasing the chromium concentration that was added. This kind of sorption curve was previously found in a study that focused on retention of arsenic (another pollutant in anionic form) using pine sawdust samples from the same origin [23]. These authors indicate that C-type curves are generally associated with the existence of a constant partition between the adsorbent surface and the equilibrium solution in the contacting layer, although another explanation can be that a proportional increase of the adsorbent surface may occur when the amount of adsorbed anion increases. It is remarkable that in the present study both pine sawdust and oak wood ash show the same type of sorption curve (C-type), while Seco-Reigosa *et al.* [23], studying As(V) sorption, found an S-type curve for oak wood ash, and a C-type curve for pine sawdust. S-type sorption curves show a not marked slope at the beginning, being more pronounced when increasing the concentration of pollutant, thus clearly differing from C-type curves. These facts indicate that, even though both pollutants are in anionic form, chromium and arsenic are subjected to sorption/desorption processes that are not coincident when using oak wood ash as adsorbent, although these processes show more similar behavior when the adsorbent material is pine sawdust.

**Figure 2:**
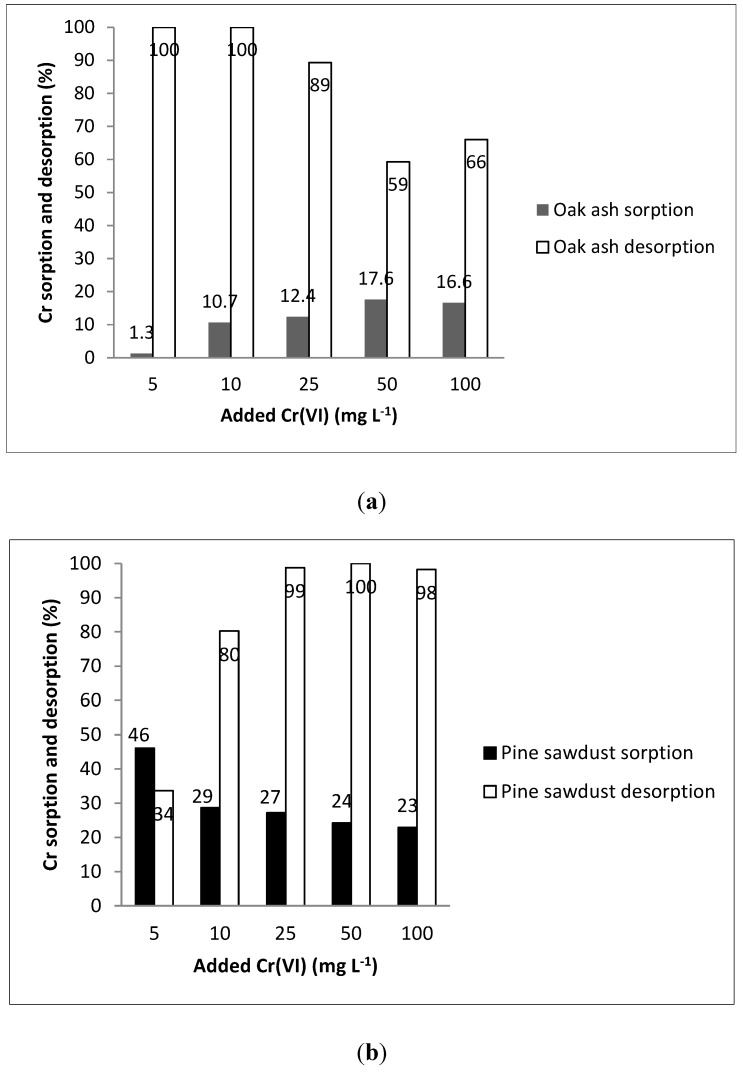
Sorption and desorption percentages corresponding to increasing Cr(VI) concentrations added to oak wood ash (**a**) or to pine sawdust (**b**).

Figure 2 shows percentage sorption (amount retained with respect to that added) and percentage desorption values (amount released with respect to that previously adsorbed) for the Cr(VI) concentrations added. Sorption was not higher than 47% on oak wood ash (Figure 2a) or on pine sawdust (Figure 2b), while desorption achieved even 98%–100% of the amounts previously adsorbed, as occurred when 5–10 mg·L^−1^ Cr(VI) were added to oak wood ash (Figure 2a), or when 25–100 mg·L^−1^ Cr(VI) were added to pine sawdust (Figure 2b). Oak wood ash retained more Cr for higher doses (50–100 mg·L^−1^), whereas pine sawdust retained more Cr when the dose was low (notably, for 5 mg·L^−1^). Also remarkable, when the highest Cr(VI) concentration (100 mg·L^−1^) was added, sorption was 22.9% on pine sawdust, but subsequent desorption was very high (98.2%), giving an overall retention not more important than that achieved on oak wood ash (sorption 16.6%, and desorption 66% of the amount previously adsorbed).

Figure 2 shows that percentage sorption results for oak wood ash and pine sawdust exhibited a clear difference in the initial stage, when the chromium concentrations added were low. Concretely, pine sawdust adsorbed more Cr when the concentrations added were low, reaching a point where percentage sorption trended to a constant value. However, percentage sorption on oak wood ash was minimal when the concentrations added were low, then increasing when they were in the range 5–10 mg·L^−1^, and finally reaching a steady level as in the case of pine sawdust.

Regarding desorption, Figure 2 shows that desorption from oak wood ash was 66% when the highest Cr(VI) dose (100 mg·L^−1^) was added, whereas it was even higher (98%) in the case of pine sawdust. Seco-Reigosa *et al.* [23] previously studied As desorption from samples corresponding to the same adsorbent materials, finding that, in the case of pine sawdust, arsenic desorption trended to decrease when the concentration added became higher, thus clearly differing from chromium behavior in the present study, where Cr suffered the highest desorption (98–100%) when the concentrations added were high (25–100 mg·L^−1^).

Other authors found that sawdust was efficient for uptake of Cr(VI), but they used spruce sawdust instead of pine sawdust [28,29]. In these cases it was suggested that the adsorption occurred on the lignin or tannin molecules in the wood residue, lignin containing the same types of functional surface groups (hydroxyl groups and phenol groups) as tannin [30,31] and Johnson *et al.* [32] showed results regarding Cr sorption for various types of sawdust, indicating great variability, although there were no data for pine sawdust.

Focusing on the variables that could explain Cr retention and release, authors such as Dong *et al.* [33] and Ucun *et al.* [25] have shown that pH is a fundamental parameter affecting the sorption process. The optimal pH for Cr(VI) sorption is between 1.0 and 2.5 [15,25,34,35,36]. This very acidic pH range causes protonation of active groups in the bio-sorbent molecules, developing positive charge and attracting the Cr(VI) species that are negatively charged at that pH (HCrO_4_^−^, Cr_2_O_7_^2−^, CrO_4_^2−^) [25]. Wang *et al.* [36] found that Cr(VI) sorption diminished when pH was >4, considering that this fact was due to competence between Cr(VI) oxyanions and OH^−^. In another study, Choppala *et al.* [2] observed low sorption for Cr(VI) anions in alkaline to slightly acidic soils. Griffin *et al.* [37] signaled that Cr(VI) exists as HCrO_4_^−^ in acidic soils and as CrO_4_^2−^ in alkaline soils, and they detected noticeable sorption of HCrO_4_^−^ but negligible sorption of CrO_4_^2−^.

In the present study, oak wood ash had a clearly alkaline pH (11.19), causing that the sorption mechanism taking place on this material have to be other than protonation in acid medium. However, in the case of pine sawdust, which had pH 4.91, interactions between positively charged functional groups of organic compounds and Cr(VI) anions can take place. Seco-Reigosa *et al.* [23] discussed the sorption of arsenic (another pollutant in anionic form) on the same sorbent materials, indicating that oak wood ash has high non-crystalline (oxalate-extracted) and total Al and Fe contents, which could have positive effects on the retention of anionic pollutants; however, the very alkaline pH value of this ash makes it difficult for variable charge compounds to be positively charged, thus causing anion sorption (including that affecting to chromium anions) to hardly take place. In such cases, cationic bridges could aid to retain anionic pollutants. Regarding pine sawdust, although it has an acid pH, its moderate or low total and amorphous Al and Fe contents did not aid to reach high chromium sorption.

**Figure 3:**
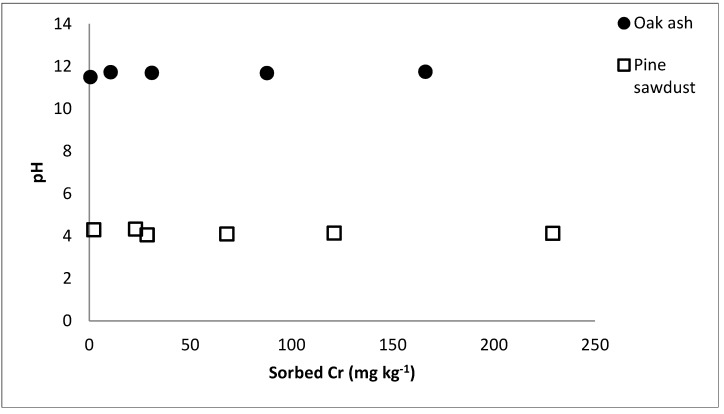
pH value in relation to the Cr adsorbed concentrations in oak wood ash and pine sawdust.

Figure 3 shows that, in the case of oak wood ash and pine sawdust, pH values hardly changed when Cr sorption increased. However, other authors observed pH increases as a function of increasing anion sorption that caused OH^−^ release, as was the case for fluoride [24,38,39] In the present study, mechanisms that do not implicate OH^−^ release, such as H and Van der Waals bindings, could be of importance in relation to Cr(VI) sorption [34].

Table 2 indicates that sorption data can be satisfactory fitted to the Freundlich and Lineal models. Vinodhini and Nilanjana [26] found good fitting to the Langmuir model when studying Cr(VI) sorption on various bio-sorbents; however, in the present study the adjustment was not possible due to very high error values associated with calculation process.

Table 3 shows that previous studies dealing with Cr(VI) sorption gave retention capacity values (*K_F_*) similar to those obtained in the present work (as is the case of anaerobic activated sludge and *Synechocystis sp.*), or lower values (as in *Scenedesmus obliquus* and *Chlorella vulgaris*), or even higher values (as in the case of pine biomass, *Rhizopus nigrificans* and *Rhizopus arrhizus*). The *K_F_* value corresponding to pine biomass was the highest in Table 3, being almost 11 times that corresponding to our pine sawdust. Regarding sorption intensity (*n*), the values referenced in Table 3 are similar to that obtained in the present work, with the exception of the higher scores showed by *Rhizopus arrhizus* and pine biomass.

**Table 2:** Constants and R^2^ coefficients corresponding to the fitting of Cr sorption data to the Freundlich and Lineal models for oak wood ash and pine sawdust.

Sorbent	Freundlich				Lineal		
	*K_F_* (L^n^ g^−1^·mg^(1−n)^)	*n*	R^2^		*a* (mg·kg^−1^)	*K_a_* (L·kg^−1^)	R^2^
Oak ash	1.920	1.117	0.990		−9.313	2.140	0.993
Pine sawdust	3.141	0.842	0.997		10.148	2.873	0.996

**Table 3:** Freundlich constants corresponding to various adsorbent materials used to retain Cr(VI) in previous studies.

Adsorbents	*K_F_*	*n*	Reference
Pine biomass	38.38	2.86	Ucun *et al.* [25]
*Rhizopus nigrificans*	12.06	0.31	Sudha-Bai and Abraham [40]
*Rhizopus arrhizus*	10.99	5.55	Prakasham *et al.* [41]
Anaerobic activated sludge	3.62	0.79	Aksu and Akpinar [42]
*Synechocystis sp.*	1.54	0.71	Cetinkaya-Donmez *et al.* [43]
*Scenedesmus obliquus*	0.68	0.70	Cetinkaya-Donmez *et al.* [43]
*Chlorella vulgaris*	0.48	0.79	Cetinkaya-Donmez *et al.* [43]

## 4. Conclusions

The oak wood ash and pine sawdust materials used in this study showed low efficacy to remove Cr from polluted media, due to their low chromium sorption capacities and high release of the previously sorbed amounts of the pollutant, which is more relevant in the case of pine sawdust. Sorption data were satisfactory fitted to the Freunlich and Lineal models, indicating that maximum sorption values are not easily predictable for these materials. These results should be taken into account to plan appropriate recycling of both by-products.
